# Indoor allergen exposure in relation to sleep health among US adults

**DOI:** 10.1016/j.jacig.2025.100441

**Published:** 2025-02-18

**Authors:** Jamie A. Murkey, Jesse Wilkerson, Paivi M. Salo, Peter S. Thorne, Darryl C. Zeldin, Chandra L. Jackson

**Affiliations:** a Epidemiology Branch, Department of Health and Human Services, National Institute of Environmental Health Sciences, National Institutes of Health, Research Triangle Park, Durham, NC; b DLH, LLC, Bethesda, Md; c Division of Intramural Research, National Institute of Environmental Health Sciences, National Institutes of Health, Research Triangle Park, NC; d Department of Occupational and Environmental Health, University of Iowa, Iowa City, Iowa; e Intramural Program, Department of Health and Human Services, National Institute on Minority Health and Health Disparities, National Institutes of Health, Bethesda, Md

**Keywords:** Allergens, indoor air quality, sleep, health inequities, social determinants of health, race factors

## Abstract

**Background:**

Common indoor allergens can accumulate within the sleep microenvironment (eg, bedding) and may contribute to poor sleep health.

**Objective:**

We sought to examine bedroom allergen exposure in relation to multiple sleep dimensions among US adults.

**Methods:**

Data for this study (N = 3399) were collected during the 2005 to 2006 National Health and Nutrition Examination Survey. Concentrations of 8 bedroom allergens were assessed and classified as elevated when levels exceeded 75th/90th percentile thresholds. Self-reported sleep measures included having trouble sleeping, any sleep disorder, snoring, and sleep medication use. Adjusting for confounders, we used Poisson regression to estimate associations between bedroom allergen exposures and sleep dimensions overall and by race/ethnicity, sex/gender, and socioeconomic status.

**Results:**

Among adults, elevated pet allergen exposure was the most prevalent (41.2%). Elevated pest allergen exposure was associated with a lower likelihood of a reported sleep disorder diagnosis (prevalence ratio [PR_overall_], 0.68; 95% CI, 0.51-0.90). For Hispanic/Latino participants, elevated pet allergen exposure was associated with having trouble sleeping (PR, 1.74; 95% CI, 1.02-2.96) and frequent snoring (PR, 1.31; 95% CI, 1.01-1.70). Elevated fungal allergen exposure was associated with any sleep disorder diagnosis among participants with moderate socioeconomic status (PR, 3.31; 95% CI, 1.21-9.10) and a higher prevalence of sleep medication use for Hispanic/Latino participants (PR, 5.72; 95% CI, 2.53-12.90; *P*_interaction_ < .01). Elevated exposures to pet (PR, 1.93; 95% CI, 1.12-3.32) and fungal (PR, 1.71; 95% CI, 1.05-2.80) allergens were also associated with being diagnosed with any sleep disorder among women.

**Conclusions:**

In a nationally representative sample of US adults, exposure to elevated levels of bedroom allergens was associated with poor sleep health, and the magnitude of the associations was generally the strongest among minoritized racial/ethnic groups and women.

The public health importance of sleep health has been underrecognized although it is essential for maintaining optimal health and well-being over one’s lifespan.^1^, ^2^, ^3^ For instance, poor sleep health is associated with adverse health conditions such as obesity, hypertension, and type 2 diabetes.^4^, ^5^, ^6^, ^7^, ^8^, ^9^, ^10^ Short sleep duration has also recently been established as a risk factor for cardiovascular disease.^11^, ^12^, ^13^, ^14^, ^15^ Unfortunately, temporal trends in the prevalence of sleep deficiencies (eg, short sleep duration) and disorders (eg, obstructive sleep apnea and insomnia)^16^, ^17^, ^18^, ^19^ have generally increased over time, especially among racially/ethnically minoritized groups that are also disproportionately burdened by the aforementioned health sequelae.^16^^,^^18^, ^19^, ^20^

Environmental allergens are hypothesized to disrupt sleep health by inducing allergic rhinitis (ie, sneezing and rhinorrhea), interfering with sleep health.^21^, ^22^, ^23^, ^24^, ^25^, ^26^, ^27^, ^28^, ^29^ Although there are burgeoning investigations of outdoor environmental contributors to unfavorable sleep health, the existing sparse evidence suggests that the indoor environment may also be important.^30^^,^^31^ For example, the sleep microenvironment—defined as the mattress, bed frame, bedding, pillows, and the air surrounding them (the breathing zone)^32^—can serve as a reservoir for relatively common indoor allergens that come from dust mites, pets, pests, and fungi.^32^^,^^33^ Presuming that the recommendation of 7 hours or more of sleep is met, at least 30% of the 24-hour day may be spent in the sleep microenvironment, potentially contributing to considerable exposure to indoor allergens.^10^^,^^11^^,^^34^, ^35^, ^36^, ^37^, ^38^

The burden of adverse environmental exposures is not distributed equally and could contribute to disparities in sleep and subsequent health outcomes. For instance, findings from a recent study suggest that trends in the prevalence of short sleep duration for US adults increased from 2004 to 2018, which was persistently the highest among non-Hispanic (NH)–Black adults.^39^ Furthermore, some evidence suggests that women are generally more likely than men to experience short sleep duration,^39^ poor sleep quality,^40^ and sleep medication use.^40^^,^^41^ Indoor allergens are also important risk factors for allergic sensitization^42^^,^^43^ and atopic diseases (eg, asthma and eczema),^42^, ^43^, ^44^, ^45^ which tend to be disproportionately experienced by racially/ethnically minoritized groups.^46^, ^47^, ^48^

Despite its importance, few studies have investigated associations between bedroom allergen exposure and sleep health, and most included only sleep duration despite there being other known and independently important dimensions of sleep health related to quality.^29^^,^^49^ Furthermore, previous studies were neither racially/ethnically diverse nor nationally representative. Herein, to our knowledge, this study is the first to address important gaps in the literature by assessing indoor allergen exposures in relation to various sleep dimensions among a nationally representative sample of racially/ethnically and socioeconomically diverse men and women in the United States. We hypothesized that the prevalence or percentage of participants with elevated exposure to indoor allergens and having trouble sleeping, frequent snoring, and any sleep disorder would be higher among adults from minoritized racial/ethnic groups compared with NH-White adults. We also hypothesized that elevated exposure to indoor allergens would be associated with having trouble sleeping, frequent snoring, any sleep disorder, and sleep medication use in the overall population and that the allergen-sleep relationship would be stronger among minoritized racial/ethnic groups compared with NH-White adults, among women compared with men, and among individuals with low socioeconomic status (SES) compared to those with higher SES.

## Methods

### Data source and study design

Data for this study were collected during the National Health and Nutrition Examination Survey (NHANES) 2005 to 2006 cycle. The NHANES is a continuous cross-sectional survey of the US noninstitutionalized civilian population conducted by the National Center for Health Statistics of the Centers for Disease Control and Prevention. It uses a complex multistage sampling design to derive a sample representative of the US population. The NHANES 2005 to 2006 cycle included an in-home dust collection component that assessed indoor allergen levels in reservoir dust samples from participants’ bedrooms and a sleep disorders questionnaire section. A detailed description of the study procedures is available in the NHANES Allergen Dust Collection Procedures Manual.^50^ Among the 10,348 participants, 6,963 had complete or partial indoor dust collection data, with 2,680 not queried on sleep disorders or indicators of poor sleep, 880 younger than 20 years, and 4 with no data on any of the analyzed indoor allergens. Our final analytic study sample consisted of 3,399 participants who had data on sleep disorders, indicators of poor sleep, and bedroom allergen levels and were aged 20 years or older. The NHANES protocols were approved by the institutional review boards of the National Center for Health Statistics and the Centers for Disease Control and Prevention and informed consent was obtained from all participants. In addition, approval for the use of nonidentifiable, publicly available NHANES data was waived by the National Institute of Environmental Health Sciences Institutional Review Board. The NHANES 2005 to 2006 unweighted response rate for the interviewed sample was 80.5%.^51^

### Exposure assessment: Indoor allergens

Indoor allergens were grouped as follows: ‘dust mite' (Der f 1 and Der p 1), ‘pest' (cockroach [Bla g 1], ‘mouse' [Mus m 1], and ‘rat' [Rat n 1]), ‘pet' (cat [Fel d 1] and dog [Can f 1]), and ‘fungal' (*Alternaria alternata* [Alt a 1]) allergens. Combined bed and bedroom floor dust samples were collected at each participant’s home using a Sanitaire Model 3683 vacuum cleaner fitted with a Mitest Dust Collector (InBio, Charlottesville, Va). An area of 0.84 m^2^ on each surface was vacuumed for 2 minutes and combined into a composite bedroom dust sample. Allergens were extracted from dust samples using endotoxin-free water containing 0.05% Tween-20 (https://wwwn.cdc.gov/nchs/data/nhanes/2005-2006/labmethods/aldust_d_met_endotoxin.pdf). Allergen concentrations for dust mite (Der f 1 and Der p 1), cockroach (Bla g 1), mouse (Mus m 1), rat (Rat n 1), cat (Fel d 1), dog (Can f 1), and the fungal allergen *Alternaria alternata* (Alt a 1) were assessed by immunoassay. Allergen exposures were considered elevated if their concentration was higher than previously defined, percentile-based (75th/90th) thresholds (0.388 μg/g_Der f 1_, 0.219 μg/g_Der p 1_, 1.778 μg/g_Bla g 1_, 0.238 μg/g_Mus m 1_, 0.013 μg/g_Rat n 1_, 6.369 μg/g_Fel d 1_, 8.472 μg/g_Can f 1_, and 0.009 μg/g_Alt a 1_).^52^ Allergen exposure in each group was considered elevated if any of the allergens in the group exceeded threshold levels.^52^ The Multiplex Array for Indoor Allergens (InBio) was used to assess each allergen apart from Bla g 1, which was assessed using ELISA. Details of the laboratory methods and quality control procedures are provided elsewhere.^53^

### Outcome assessment: Sleep disorders and poor sleep indicators

Sleep disorders and sleep quality measures were assessed using questionnaires. Participants self-reported doctor-diagnosed sleep disorders (“Have you ever been told by a doctor or other health professional that you have a sleep disorder?”) and self-reported indicators of sleep quality, such as trouble sleeping (“Have you ever told a doctor or other health professional that you have trouble sleeping?”), frequent snoring (“In the past 12 months, how often did you snore while you were sleeping?” [≥3 nights/wk]), and frequent sleep medication use (“In the past month, how often did you take sleeping pills or other medication to help you sleep?” [≥5 nights/mo]).

### Potential modification assessment: Race/ethnicity, sex/gender, and SES

Sociodemographic characteristics were selected as potential modification *a priori* on the basis of existing literature, including self-identified race/ethnicity (NH-White, NH-Black, Hispanic/Latino, and other race), sex/gender (men and women), and SES (poverty-income ratio [PIR] calculated as the ratio of household income to the poverty threshold and categorized in the following levels: <1.0 [below poverty], 1.0-1.85 [eligible for benefits of the Special Supplemental Nutrition Program for Women, Infants, and Children], and >1.85 [ineligible for such benefits]).^54^, ^55^, ^56^, ^57^, ^58^ On the basis of PIR, SES was defined as low (PIR < 1.0), moderate (PIR = 1.0-1.85), and high (PIR > 1.85).

### Potential confounders

Potential confounders—determined on the basis of previous literature—included age (in years), sex/gender (men and women), self-identified race/ethnicity (NH-White, NH-Black, Hispanic/Latino, and other race), SES (low, moderate, and high), housing type (mobile home or trailer, 1-family home, and multifamily housing), household size, flooring type (any carpeting or smooth surface), alcohol consumption, and smoking status (current, former, and never).^54^, ^55^, ^56^, ^57^, ^58^ Data on body mass index (BMI) were ascertained via physical examination, and data on sensitization to inhalant allergens were determined via analysis of blood serum samples.^48^ Age was dichotomized as 20 to 49 years and 50 years or more.^59^^,^^60^ Household size was categorized as 1 to 2 occupants, 3 to 4 occupants, and 5 or more occupants.^57^^,^^61^ Alcohol consumption was categorized as 0 drinks/wk (non-drinker), 1 to 6 drinks/wk (light drinker), and more than or equal to 7 drinks/wk (heavy drinker).^54^^,^^62^ BMI was calculated as weight in kilograms divided by height in meters squared and was categorized into different levels (<18.5 kg/m^2^ [underweight], 18.5 to <25 kg/m^2^ [normal], 25 to <30 kg/m^2^ [overweight], and ≥30 kg/m^2^ [obese]).^54^^,^^63^ Sensitization to inhalant allergens was dichotomized as a serum specific IgE level of greater than or equal to 0.35 kU/L for any of 15 inhalant allergens measured by the NHANES.^48^ Season of data collection was dichotomized in 6-month intervals (November-April and May-October), and presence of furry pets in home was determined via questionnaire response.

### Statistical analysis

We summarized participants’ sociodemographic characteristics, health behaviors, and clinical characteristics using descriptive statistics overall and stratified by race/ethnicity category. Bivariate differences in the prevalence of dust allergens, poor sleep indicators, and potential confounders across racial/ethnic groups were evaluated using the chi-square test. Adjusting for age, sex/gender, race/ethnicity, PIR, housing type, household size, flooring type, alcohol consumption, smoking status, BMI, sensitization to inhalant allergens, season of data collection, and presence of furry pets in home, we used Poisson regression with robust variance to estimate prevalence ratios (PRs) and 95% CIs between elevated allergen levels and each sleep measure. We tested for interactions by including multiplicative interaction terms (ie, race/ethnicity × bedroom dust allergen groups, sex/gender × bedroom allergen groups, and SES × bedroom allergen groups) and used the resulting Wald chi-square *P* values to assess potential effect modification in our statistical models between bedroom allergen exposure and poor sleep health indicators. In addition, we performed subgroup analyses stratifying by race/ethnicity, sex/gender, and SES. Descriptive analyses were performed using SAS software (version 9.4; SAS Institute, Cary, NC), and Poisson modeling was performed using SUDAAN (version 11.0.3; RTI International, Research Triangle Park, NC). All analyses accounted for the complex survey design using sampling weights of the NHANES to obtain nationally representative estimates. *P* values less than .05 were considered to be statistically significant for descriptive and marginal regression analyses, and *P* values less than .10 were considered to be statistically significant for the assessment of interaction terms.

## Results

### Study population

Among the 3399 eligible participants, most were aged 20 to 49 years (59.3%), were women (51.9%), and lived in a one-family (65.8%) as opposed to a multifamily (26.6%) home (Table I). Most participants reported living in smaller households (1-2 individuals [47.5%]) compared with medium (3-4 individuals [36.0%]) or larger (≥5 individuals [16.5%]) households. Most participants were former or current smokers (50.8%) and had carpeting in their bedrooms (91.4%). Slightly more than half the participants (51.0%) reported frequent snoring and 25.3% reported trouble sleeping to their doctors, whereas doctor-diagnosed sleep orders (7.4%) and frequent use of sleep medication were less prevalent among participants. There were statistically significant racial/ethnic disparities in having trouble sleeping, with the prevalence ranging from 12.9% for Hispanic/Latino participants to 27.9% for NH-White participants (*P* < .001). Racial/ethnic differences were observed in terms of the prevalence of frequent sleep medication use (range, 4.2%_Hispanic/Latino_ to 13.0%_Other race_; *P* < .001). No significant racial/ethnic differences were observed for doctor-diagnosed sleep disorder nor frequent snoring. The prevalence of elevated dust allergen exposure was 38.5% for dust mites, 24.4% for pests, 41.2% for pets, and 10.0% for fungal allergens. Racial/ethnic differences were observed for the pest (range, 19.3%_Other race_ to 32.2%_Hispanic/Latino_; *P* = .001), pet (range, 10.6%_NH-Black_ to 50.1%_NH-White_; *P* < .001), and fungal (range, 2.8%_NH-Black_ to 12.1%_NH-White_; *P* < .001) allergens. However, no differences were observed for the dust mite allergens (*P* = .957) (Table I).

**Table I:** Sociodemographic characteristics, health behaviors, and clinical characteristics among US adults by race/ethnicity, NHANES 2005-2006 (N = 3399)

Characteristics	Weighted %∗					*P* value
	Overall (N = 3399)	NH-White (N = 1601)	NH-Black (N = 825)	Hispanic/Latino (N = 831)	Other race (N = 142)	
*Participant characteristics*						
Age (y)						**<.001**
20-49	59.3	54.9	65.0	78.0	66.2	
≥50	40.7	45.1	35.0	22.0	33.8	
Sex/gender						.091
Men	48.1	48.5	44.9	50.8	44.7	
Women	51.9	51.5	55.13	49.2	55.3	
BMI						**<.001**
Underweight	1.8	2.0	1.3	1.3	0.9	
Normal	30.7	31.5	23.2	26.6	43.6	
Overweight	31.6	31.7	29.2	36.6	25.0	
Obese	36.0	34.9	46.3	35.5	30.6	
PIR						**<.001**
<1.0	11.8	8.0	20.3	28.4	11.5	
1.0-1.85	17.9	15.1	24.5	30.3	16.4	
>1.85	70.3	77.0	55.2	41.3	72.1	
Sensitization to inhalant allergens	42.9	40.3	53.2	44.6	52.5	**<.001**
Season of collection						**<.001**
November-April	41.1	35.1	54.8	65.6	40.5	
May-October	58.9	64.9	45.2	34.4	59.5	
*Housing characteristics*						
Type of home						**<.001**
Mobile home or trailer	7.6	7.6	7.0	7.8	8.0	
One-family house (detached)	65.8	70.6	50.6	54.4	59.0	
Multifamily housing	26.6	21.8	42.4	37.8	33.0	
Household size						**<.001**
1-2	47.5	53.9	42.2	16.3	39.0	
3-4	36.0	34.1	36.5	45.1	41.5	
≥5	16.5	12.0	21.3	38.6	19.5	
Flooring type						**<.001**
Any carpet/rug	91.4	92.9	90.6	84.8	88.7	
Smooth surface	8.6	7.1	9.4	15.2	11.3	
Furry pets in home	50.2	58.7	19.2	32.4	41.6	**<.001**
*Health behaviors*						
Alcohol consumption						**<.001**
Nondrinker	35.7	32.5	51.3	40.8	37.8	
Light drinker	48.2	49.4	38.0	47.2	54.3	
Heavy drinker	16.1	18.1	10.7	12.1	7.8	
Smoking status						**<.001**
Never smoker	49.2	43.8	60.0	65.8	62.5	
Former smoker	25.2	28.8	15.3	15.9	18.3	
Current smoker	25.6	27.4	24.8	18.3	19.2	
*Allergen exposure*						
Elevated dust mite allergen exposure	38.5	38.0	38.7	39.9	41.5	.957
Elevated pest allergen exposure	24.4	22.7	29.4	32.2	19.3	**.001**
Elevated pet allergen exposure	41.2	50.1	10.6	18.7	35.3	**<.001**
Elevated fungal allergen exposure	10.0	12.1	2.8	3.7	10.9	**<.001**
Elevated endotoxin exposure	8.2	7.2	11.5	11.7	6.8	.057
*Poor sleep indicators*						
Doctor-diagnosed sleep disorder	7.4	7.8	6.5	5.3	8.9	.506
Has trouble sleeping	25.3	27.9	21.2	12.9	26.1	**<.001**
Frequent snoring	51.0	51.2	51.4	50.4	49.3	.952
Frequent sleep medication use	9.8	10.9	6.5	4.2	13.0	**<.001**

*P* values in boldface indicate statistically significant associations.

∗ Weighted to account for the complex multistage sampling design of the NHANES.

### Indoor allergens in relation to sleep disturbances, overall

**Fig 1:**
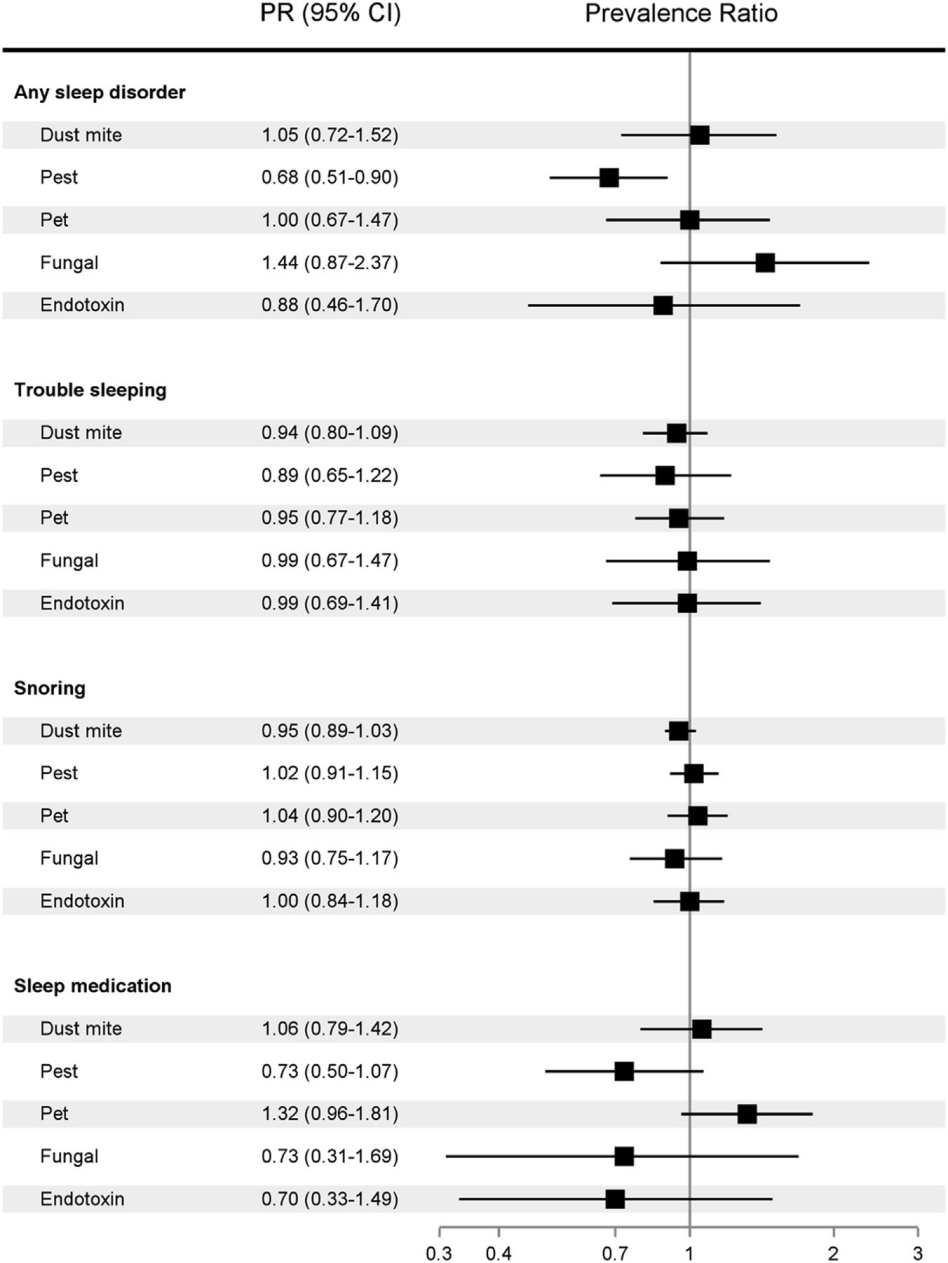
Associations between bedroom allergen and endotoxin exposures and poor sleep indicators, NHANES 2005-2006 (N = 3399).

Elevated pest allergen exposure was associated with being less likely to be diagnosed with any sleep disorder (PR, 0.68; 95% CI, 0.51-0.90) (Fig 1). No statistically significant associations were observed between other bedroom allergen and endotoxin exposures and poor sleep indicators, overall (see Tables E1 and E2).

### Indoor allergens in relation to sleep disturbances, by race/ethnicity

**Fig 2:**
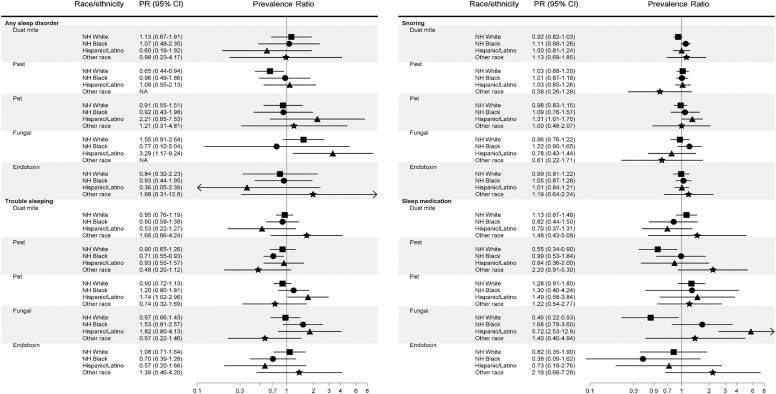
Associations between bedroom allergen and endotoxin exposures and poor sleep indicators by race/ethnicity, NHANES 2005-2006 (N = 3399).

Analyses by race/ethnicity identified associations not shown to be significant in the overall population. For Hispanic/Latino participants, elevated pet allergen was associated with having trouble sleeping (PR, 1.74; 95% CI, 1.02-2.96) and frequent snoring (PR, 1.31; 95% CI, 1.01-1.70) (Fig 2). Elevated fungal allergen exposure was also significantly associated with being diagnosed with any sleep disorder for Hispanic/Latino participants (PR, 3.29; 95% CI, 1.17-9.24). Elevated pest exposure was associated with a lower prevalence of any sleep disorder diagnosis for NH-White participants (PR, 0.65; 95% CI, 0.44-0.94) and a lower prevalence of having trouble sleeping among NH-Black participants (PR, 0.71; 95% CI, 0.55-0.93). Effect modification of allergen exposures and sleep health by race/ethnicity was primarily observed for frequent sleep medication use, having significant interactions with race/ethnicity for dust mite, pest, and fungal allergens. The most disparate was effect modification between race/ethnicity and bedroom allergen exposure in associations between fungal allergen exposure and frequent sleep medication use for participants identifying as NH-White (PR, 0.46; 95% CI, 0.22-0.93) and Hispanic/Latino (PR, 5.72; 95% CI, 2.53-12.90; *P*_interaction_ < .01) (see Tables E1 and E3 in this article’s Online Repository at www.jaci-global.org).

### Indoor allergens in relation to sleep disturbances, by sex/gender

**Fig 3:**
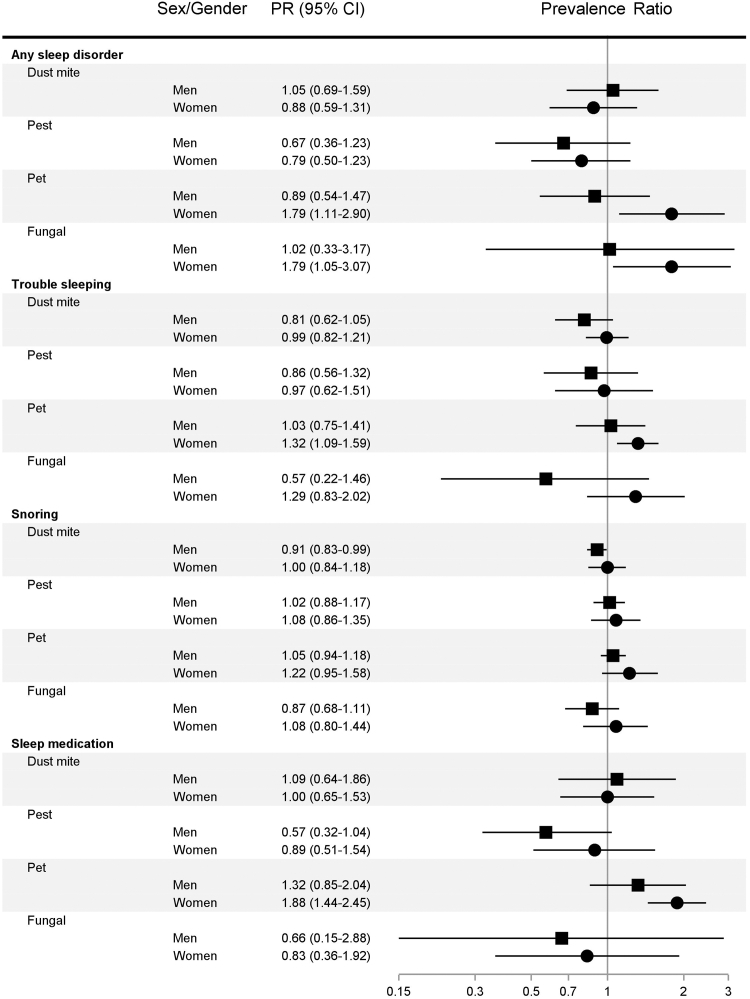
Associations between bedroom allergen and endotoxin exposures and poor sleep indicators by sex/gender, NHANES 2005-2006 (N = 3399).

Among women, exposures to elevated levels of pet (PR, 1.93; 95% CI, 1.12-3.32) and fungal (PR, 1.71; 95% CI, 1.05-2.80) allergens were associated with having any sleep disorder (Fig 3). For men, the only significant association between allergen exposure and sleep health was an 8% lower prevalence in frequent snoring among those with elevated dust mite allergen exposure (PR, 0.92; 95% CI, 0.84-0.99). The association between pet allergen exposure and frequent sleep medication use was comparable between women (PR, 1.29; 95% CI, 0.91-1.82) and men (PR, 1.31; 95% CI, 0.68-2.53) (*P*_interaction_ = .062; see Tables E2 and E3). However, the magnitude of the association between fungal allergen exposure and having trouble sleeping was higher among women than among men (PR, 1.23 vs 0.55; *P*_interaction_ = .093).

### Indoor allergens in relation to sleep disturbances, by SES

**Fig 4:**
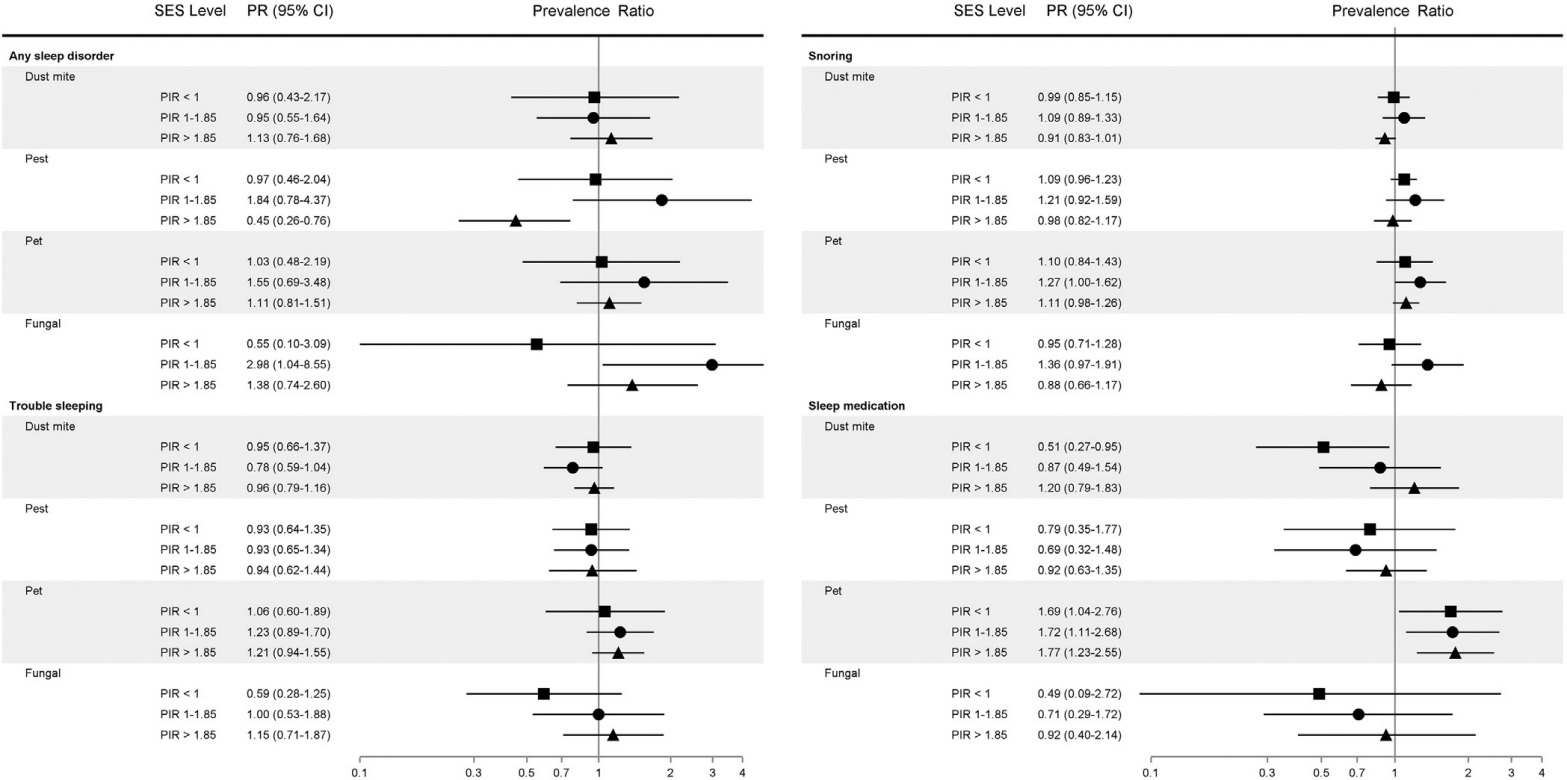
Associations between bedroom allergen and endotoxin exposures and poor sleep indicators by SES, NHANES 2005 to 2006 (N = 3399).

Elevated fungal (PR, 3.31; 95% CI, 1.21-9.10) and pet (PR, 2.60; 95% CI, 1.33-5.07) allergen exposures were associated with any sleep disorder diagnosis among participants with moderate SES (Fig 4). Conversely, elevated pest allergen exposure was associated with being less likely to be diagnosed with any sleep disorder among those with higher SES (PR, 0.43; 95% CI, 0.25-0.72).

## Discussion

In this nationally representative study of the US population, we investigated exposure to indoor allergens in relation to various sleep dimensions among adults. We found that elevated exposure to pest allergens was associated with a lower prevalence of being diagnosed with any sleep disorder in the overall study sample. Associations between elevated exposure to pet allergens and having trouble sleeping were the strongest among Hispanic/Latino adults. Elevated pest exposure was associated with a lower prevalence of being diagnosed with any sleep disorder among NH-White participants and with a lower prevalence of having trouble sleeping among NH-Black participants. In addition, elevated fungal allergen exposure was associated with a higher prevalence of being diagnosed with any sleep disorder among Hispanic/Latino adults and frequent sleep medication use among NH-White and Hispanic/Latino participants. Elevated exposures to pet and fungal allergens were associated with being diagnosed with any sleep disorder among women. In addition, elevated fungal and pet allergen exposures were associated with being diagnosed with any sleep disorder among those with moderate SES, whereas elevated pest allergen exposure was associated with being less likely to be diagnosed with any sleep disorder among those with higher SES.

Mechanisms underlying differences in the magnitude of these associations are not well understood. For instance, conflicting evidence in the literature has described associations between differential pet ownership and allergic sensitization. Some evidence suggests that early-life pet exposure may be protective against allergen sensitization. For example, previous studies reported that cat and dog exposure among children before age 1 year is associated with reduced allergen sensitization.^64^^,^^65^ Considering that cat and dog exposure may play a role in reducing risk for allergen sensitization *in utero*, and that NH-Black and Hispanic/Latino individuals are less likely than those who are NH-White to own a cat or dog,^66^^,^^67^ differential pet ownership by race/ethnicity may help explain our findings. However, a separate study reported that at age 2 years, NH-Black children were more likely than NH-White children to test positive for allergic disease–related outcomes, even after adjusting for maternal indoor pet exposure during pregnancy, along with sociodemographic characteristics (eg, household income).^68^ Thus, the role of indoor allergen exposure in the development of allergic sensitization and disease is complex and warrants further investigation.

Disparities in unfavorable sleep health following exposure to elevated levels of indoor allergens were also observed after stratifying by sex/gender. Associations between elevated pet and fungal allergen exposure and having any sleep disorder were observed among women but not men. Our findings are consistent with previous research demonstrating that cat or dog ownership among women compared with men is associated with a higher odds of having trouble with sleeping.^69^ Furthermore, our finding that elevated pest allergen exposure was associated with a lower prevalence of being diagnosed with a sleep disorder corroborates results reported by a 2023 study that observed a positive relationship of pediatric sleep-disordered breathing with indoor pest allergen exposure, SES, and environmental tobacco smoke.^70^ Although we did not delineate differences in associations between trouble with sleeping by pet allergen exposure type in this study, some evidence suggests that associations are even stronger for dog versus cat allergen exposure among women.^69^ Subgroup analyses by sociodemographic characteristics also yielded additional findings not observed in the overall study sample. For example, elevated fungal allergen exposure was associated with a sleep disorder diagnosis among Hispanic/Latino adults, women, and those with moderate SES. Although causal mechanisms explaining these findings remain unclear, associations have been identified between indoor fungal exposure and the onset^45^^,^^71^ and exacerbation^45^^,^^72^ of allergic respiratory conditions such as hay fever, allergic rhinitis, and asthma. Considering that risk for disordered sleeping is associated with asthma,^73^ for which racial/ethnic disparities exist among racially/ethnically minoritized groups,^74^ certain allergic respiratory diseases may mediate the relationship.

Although indoor fungal exposure is relatively common in US households,^33^^,^^44^ a potential explanation for why differences were observed among socioeconomically disadvantaged and minoritized racial/ethnic subgroups may be based on variation of indoor household mold and dampness levels, which increases the likelihood of concomitant coexposures.^33^^,^^75^^,^^76^ For example, dust mite allergen level has been positively associated with indoor household mold.^75^^,^^76^ Previous literature has found that homes that were older^75^^,^^76^ and without air conditioning^76^ tend to promote mold growth, which has been associated with lower SES and being NH-Black.^76^ Furthermore, increases in mold and other indoor pollutants may be facilitated through the transfer of environmental microbiota from outdoor to indoor environments via pets. The measurement of mold allergens may also be a proxy for microbe-associated molecular patterns in mold spores, especially 1,3-β-d-glucans, which are inflammatory and likely induce disordered sleep.^77^^,^^78^ However, findings from our study did not observe statistically significant associations between endotoxin exposure and poor sleep. Additional findings from our study indicate that NH-Black adults carried the greatest burden of sensitization to inhalant allergens, potentially indirectly resulting in more frequent sleep medication use to ease discomfort and agitation interfering with sleep.^21^, ^22^, ^23^, ^24^, ^25^, ^26^, ^27^, ^28^ Results indicating significant interactions for frequent sleep medication use with dust mite, pest, and fungal allergens being the strongest among minoritized racial/ethnic groups corroborate findings from previous literature describing associations between pet ownership and sleep dissatisfaction,^79^ insufficient sleep,^79^ and overall sleep quality.^69^ In addition, unfavorable sleep patterns experienced by pet owners, partially attributed to pet allergen exposure, may necessitate more frequent sleep medication use.^69^

Limitations of this study warrant consideration. First, the NHANES data used for our study were based on a cross-sectional study design, preventing causal inference. Also, selection bias may have been introduced after excluding individuals not meeting the inclusion criteria used for this study (see Table E4 in this article’s Online Repository at www.jaci-global.org). Furthermore, participants self-reported doctor-diagnosed sleep disorders and indicators of sleep quality (eg, trouble sleeping), which may result in misclassification when compared with objective measures. It is worth noting, however, that measurement error between self-reported and objectively measured sleep duration was comparable and nondifferential across racial/ethnic groups.^80^ Furthermore, individuals from other racially/ethnically minoritized groups not identifying as NH-Black or Hispanic/Latino were aggregated, precluding a separate investigation of differences in allergen exposure and sleep patterns among those groups. In addition, although our study findings indicate that racial/ethnic and sex/gender disparities in associations between indoor allergen exposures and unfavorable sleep patterns exist, we did not investigate the intersectionality of them combined.

Despite these limitations, our study also has important strengths. To our knowledge, this study is the first to assess bedroom allergen exposures in relation to multiple sleep dimensions among a racially/ethnically diverse, nationally representative US sample of adults. We used a nationally representative and racially/ethnically diverse sample, making the investigation of allergen-sleep associations by race/ethnicity permissible. Also, indoor allergens were objectively measured and did not rely on participant self-reports. In addition, we analyzed multiple dimensions of sleep, whereas most previous studies included mainly sleep duration alone. Future longitudinal studies that are larger and racially/ethnically diverse are needed to investigate mechanisms or pathways explaining disparities between indoor allergen exposure and multiple sleep dimensions. These studies should build on our findings by exploring the potential influence of intersectionality (race/ethnicity and sex/gender) on associations between indoor environmental allergen exposure and multiple sleep dimensions.

We found that elevated indoor allergen exposure was associated with multiple dimensions of sleep in a nationally representative study. Results from our study also highlighted sleep health disparities and that disparate exposure to indoor allergens may more generally exacerbate poor sleep more strongly among women and racially/ethnically minoritized groups. Considering that insufficient sleep and poor sleep quality have been associated with higher susceptibility to morbidity and mortality, public health interventions may eventually include addressing environmental allergens once future studies establish a causal relationship.

**Clinical implications**

Mitigating bedroom allergen exposure may alleviate sleep health disparities.

## Disclosure statement

This work was funded, in part, by the Intramural Program at the National Institute of Environmental Health Sciences, National Institutes of Health (grant no. Z1A ES103325 to C.L.J. and grant no. Z01 ES025041 to D.C.Z.), and by the Intramural Research Program at the National Institute on Minority Health and Health Disparities, National Institutes of Health.

Disclosure of potential conflict of interest: The authors declare that they have no relevant conflicts of interest.
