# Supplementary material for: Indoor allergen exposure in relation to sleep health among US adults
Source: J Allergy Clin Immunol Glob. 2025 Feb 18;4(2):100441. doi: 10.1016/j.jacig.2025.100441 (PMC11978379; doi:10.1016/j.jacig.2025.100441)
Supplement: Supplementary Table 1-4 [file mmc1.docx]

# Supplemental Table 1. Associations between indoor allergen and endotoxin exposures and poor sleep indicators overall and stratified by race/ethnicity, NHANES 2005-2006 (N=3,399)

|  | **Overall** | **NH-White** | **NH-Black** | **Hispanic/Latino** | **Other Race** |
| --- | --- | --- | --- | --- | --- |
|  | **PR (95% CI)** | **PR (95% CI)** | **PR (95% CI)** | **PR (95% CI)** | **PR (95% CI)** |
| **Any sleep disorder** |  |  |  |  |  |
| Dust mite | 1.05 (0.72-1.52) | 1.13 (0.67-1.91) | 1.07 (0.48-2.35) | 0.60 (0.19-1.92) | 0.98 (0.23-4.17) |
| Pest | **0.68 (0.51-0.90)** | **0.65 (0.44-0.94)** | 0.96 (0.49-1.86) | 1.08 (0.55-2.13) | NA |
| Pet | 1.00 (0.67-1.47) | 0.91 (0.55-1.51) | 0.92 (0.43-1.98) | 2.21 (0.65-7.53) | 1.21 (0.31-4.81) |
| Fungal | 1.44 (0.87-2.37) | 1.55 (0.91-2.64) | 0.77 (0.12-5.04) | **3.29 (1.17-9.24)** | NA |
| Endotoxin | 0.88 (0.46-1.70) | 0.84 (0.32-2.23) | 0.93 (0.44-1.95) | 0.36 (0.05-2.39) | 1.98 (0.31-12.83) |
| **Trouble sleeping** |  |  |  |  |  |
| Dust mite | 0.94 (0.80-1.09) | 0.95 (0.76-1.19) | 0.90 (0.59-1.38) | 0.53 (0.22-1.27) | 1.68 (0.66-4.24) |
| Pest | 0.89 (0.65-1.22) | 0.90 (0.65-1.26) | **0.71 (0.55-0.93)** | 0.93 (0.55-1.57) | 0.48 (0.20-1.12) |
| Pet | 0.95 (0.77-1.18) | 0.90 (0.72-1.13) | 1.20 (0.80-1.81) | **1.74 (1.02-2.96)** | 0.74 (0.32-1.69) |
| Fungal | 0.99 (0.67-1.47) | 0.97 (0.66-1.43) | 1.53 (0.91-2.57) | 1.82 (0.80-4.13) | 0.57 (0.22-1.46) |
| Endotoxin | 0.99 (0.69-1.41) | 1.08 (0.71-1.64) | 0.70 (0.39-1.28) | 0.57 (0.20-1.66) | 1.39 (0.46-4.20) |
| **Snoring** |  |  |  |  |  |
| Dust mite | 0.95 (0.89-1.03) | 0.92 (0.82-1.03) | 1.11 (0.98-1.26) | 1.00 (0.81-1.24) | 1.13 (0.69-1.85) |
| Pest | 1.02 (0.91-1.15) | 1.03 (0.88-1.20) | 1.01 (0.87-1.18) | 1.03 (0.85-1.26) | 0.58 (0.26-1.29) |
| Pet | 1.04 (0.90-1.20) | 0.98 (0.83-1.15) | 1.09 (0.76-1.57) | **1.31 (1.01-1.70)** | 1.00 (0.48-2.07) |
| Fungal | 0.93 (0.75-1.17) | 0.96 (0.76-1.22) | 1.22 (0.90-1.65) | 0.78 (0.43-1.44) | 0.61 (0.22-1.71) |
| Endotoxin | 1.00 (0.84-1.18) | 0.99 (0.81-1.22) | 1.05 (0.87-1.28) | 1.01 (0.84-1.21) | 1.19 (0.64-2.24) |
| **Sleep medication** |  |  |  |  |  |
| Dust mite | 1.06 (0.79-1.42) | 1.13 (0.87-1.48) | 0.82 (0.44-1.50) | 0.70 (0.37-1.31) | 1.48 (0.43-5.09) |
| Pest | 0.73 (0.50-1.07) | **0.55 (0.34-0.90)** | 0.99 (0.53-1.84) | 0.84 (0.36-2.00) | 2.20 (0.91-5.30) |
| Pet | 1.32 (0.96-1.81) | 1.28 (0.91-1.80) | 1.30 (0.40-4.24) | 1.49 (0.58-3.84) | 1.22 (0.54-2.77) |
| Fungal | 0.73 (0.31-1.69) | **0.46 (0.22-0.93)** | 1.68 (0.78-3.60) | **5.72 (2.53-12.94)** | 1.40 (0.40-4.94) |
| Endotoxin | 0.70 (0.33-1.49) | 0.82 (0.35-1.90) | 0.38 (0.09-1.62) | 0.73 (0.19-2.76) | 2.19 (0.66-7.28) |

Abbreviations: NHANES = National Health and Nutrition Examination Survey; NH = non-Hispanic

# Supplemental Table 2. Associations between indoor allergen and endotoxin exposures and poor sleep indicators stratified by sex/gender and socioeconomic status, NHANES 2005-2006 (N=3,399)

|  | **Men** | **Women** | **PIR < 1** | **PIR 1-1.85** | **PIR > 1.85** |
| --- | --- | --- | --- | --- | --- |
|  | **PR (95% CI)** | **PR (95% CI)** | **PR (95% CI)** | **PR (95% CI)** | **PR (95% CI)** |
| **Any sleep disorder** |  |  |  |  |  |
| Dust mite | 1.06 (0.69-1.64) | 0.89 (0.61-1.28) | 1.01 (0.53-1.90) | 0.90 (0.53-1.53) | 1.15 (0.78-1.71) |
| Pest | 0.66 (0.36-1.19) | 0.77 (0.50-1.20) | 1.00 (0.49-2.02) | 1.77 (0.77-4.07) | **0.43 (0.25-0.72)** |
| Pet | 0.69 (0.35-1.34) | **1.93 (1.12-3.32)** | 1.00 (0.45-2.26) | **2.60 (1.33-5.07)** | 0.84 (0.52-1.35) |
| Fungal | 0.99 (0.32-3.09) | **1.71 (1.05-2.80)** | 0.65 (0.15-2.91) | **3.31 (1.21-9.10)** | 1.32 (0.70-2.46) |
| Endotoxin | 0.97 (0.35-2.73) | 0.79 (0.27-2.28) | 0.62 (0.22-1.76) | 1.21 (0.45-3.28) | 1.11 (0.44-2.77) |
| **Trouble sleeping** |  |  |  |  |  |
| Dust mite | 0.81 (0.63-1.05) | 1.00 (0.83-1.22) | 0.98 (0.69-1.37) | 0.81 (0.61-1.07) | 0.97 (0.81-1.15) |
| Pest | 0.85 (0.57-1.27) | 0.94 (0.60-1.48) | 0.91 (0.62-1.35) | 0.94 (0.65-1.34) | 0.91 (0.61-1.37) |
| Pet | 0.86 (0.59-1.27) | 1.04 (0.81-1.33) | 0.83 (0.49-1.41) | 1.04 (0.62-1.77) | 0.96 (0.68-1.36) |
| Fungal | 0.55 (0.22-1.41) | 1.23 (0.80-1.89) | 0.62 (0.31-1.27) | 0.96 (0.51-1.80) | 1.09 (0.67-1.77) |
| Endotoxin | 0.74 (0.40-1.36) | 1.15 (0.73-1.83) | 0.64 (0.40-1.03) | 1.14 (0.52-2.49) | 1.09 (0.68-1.75) |
| **Snoring** |  |  |  |  |  |
| Dust mite | **0.92 (0.84-0.99)** | 1.01 (0.84-1.21) | 0.99 (0.84-1.18) | 1.10 (0.91-1.33) | 0.92 (0.84-1.02) |
| Pest | 1.00 (0.87-1.16) | 1.06 (0.85-1.33) | 1.08 (0.95-1.24) | 1.17 (0.91-1.51) | 0.95 (0.79-1.15) |
| Pet | 0.97 (0.82-1.16) | 1.14 (0.87-1.48) | 1.08 (0.81-1.44) | 1.18 (0.92-1.52) | 0.99 (0.85-1.15) |
| Fungal | 0.86 (0.67-1.10) | 1.03 (0.75-1.43) | 0.95 (0.71-1.27) | 1.30 (0.90-1.87) | 0.84 (0.62-1.14) |
| Endotoxin | 0.91 (0.69-1.19) | 1.05 (0.77-1.42) | 0.78 (0.47-1.29) | 1.31 (0.96-1.80) | 0.99 (0.75-1.31) |
| **Sleep medication** |  |  |  |  |  |
| Dust mite | 1.09 (0.65-1.83) | 1.02 (0.68-1.53) | **0.53 (0.28-0.98)** | 0.96 (0.56-1.66) | 1.18 (0.81-1.72) |
| Pest | 0.58 (0.31-1.07) | 0.85 (0.51-1.42) | 0.80 (0.38-1.70) | 0.71 (0.32-1.60) | 0.87 (0.61-1.25) |
| Pet | 1.31 (0.68-2.53) | 1.29 (0.91-1.82) | 1.18 (0.67-2.07) | 1.07 (0.64-1.79) | 1.42 (0.94-2.15) |
| Fungal | 0.63 (0.14-2.92) | 0.77 (0.33-1.81) | 0.56 (0.12-2.65) | 0.63 (0.28-1.40) | 0.83 (0.33-2.08) |
| Endotoxin | 0.54 (0.25-1.15) | 0.78 (0.30-2.07) | 0.32 (0.06-1.68) | 1.25 (0.52-3.03) | 0.73 (0.24-2.14) |

Abbreviations: NHANES = National Health and Nutrition Examination Survey; PIR = Poverty Income Ratio

# Supplemental Table 3. Interaction P-value matrix for models of indoor

# allergen and endotoxin exposures and poor sleep indicators

|  | **Race/ethnicity** | **Sex/gender** | **Socioeconomic status** |
| --- | --- | --- | --- |
|  | **P_int_-value** | **P_int_-value** | **P_int_-value** |
| **Any sleep disorder** |  |  |  |
| Dust mite | 0.725 | 0.229 | 0.611 |
| Pest | 0.779 | 0.664 | **0.040** |
| Pet | 0.497 | 0.108 | 0.635 |
| Fungal | 0.312 | 0.517 | 0.334 |
| Endotoxin | 0.192 | 0.883 | 0.501 |
| **Trouble sleeping** |  |  |  |
| Dust mite | 0.326 | 0.256 | 0.784 |
| Pest | 0.841 | 0.832 | 0.864 |
| Pet | 0.550 | 0.203 | 0.997 |
| Fungal | 0.369 | 0.093 | 0.263 |
| Endotoxin | 0.152 | 0.300 | 0.233 |
| **Snoring** |  |  |  |
| Dust mite | 0.165 | 0.267 | 0.299 |
| Pest | 0.717 | 0.731 | 0.360 |
| Pet | 0.416 | 0.662 | 0.350 |
| Fungal | 0.315 | 0.312 | 0.196 |
| Endotoxin | 0.955 | 0.531 | 0.092 |
| **Sleep medication** |  |  |  |
| Dust mite | 0.092 | 0.839 | 0.161 |
| Pest | 0.064 | 0.092 | 0.567 |
| Pet | 0.824 | 0.062 | 0.717 |
| Fungal | **0.008** | 0.548 | 0.880 |
| Endotoxin | 0.327 | 0.288 | 0.325 |

Supplemental Table 4. Sociodemographic characteristics, health behaviors, and clinical characteristics among US adults (age 20+) by exclusion criteria, NHANES 2005-2006 (N=4,979)

|  | **Included**  **n=3,399 (67.1%)** | **Excluded**  **n=1,580 (32.9%)** |
| --- | --- | --- |
|  | Weighted %^1^ | Weighted %^2^ |
| **Participant characteristics** |  |  |
| Age (years) |  |  |
| 20 – 49 | 59.3 | 54.8 |
| 50+ | 40.7 | 45.2 |
| Sex/gender |  |  |
| Men | 48.1 | 46.4 |
| Women | 51.9 | 53.6 |
| Race/ethnicity |  |  |
| NH-White | 71.5 | 76.3 |
| NH-Black | 11.4 | 9.0 |
| Hispanic/Latino | 11.2 | 10.1 |
| Other race | 5.9 | 4.6 |
| Body mass index |  |  |
| Underweight | 1.8 | 2.0 |
| Normal | 30.7 | 32.4 |
| Overweight | 31.6 | 35.2 |
| Obese | 36.0 | 30.4 |
| Poverty income ratio |  |  |
| < 1.0 | 11.8 | 8.4 |
| 1.0 – 1.85 | 17.9 | 12.8 |
| 1.85+ | 70.3 | 78.7 |
| Sensitization to inhalant allergens | 42.9 | 39.4 |
| Season of Collection |  |  |
| November-April | 41.1 | 40.2 |
| May-October | 58.9 | 59.8 |
| **Housing characteristics** |  |  |
| Type of home |  |  |
| Mobile home or trailer | 7.6 | 6.1 |
| One family house (detached) | 65.8 | 74.3 |
| Multi-family housing | 26.6 | 19.6 |
| Household size |  |  |
| 1 – 2 | 47.5 | 54.0 |
| 3 – 4 | 36.0 | 33.2 |
| 5+ | 16.5 | 12.8 |
| Furry pets in home | 50.2 | 51.4 |
| **Health Behaviors** |  |  |
| Alcohol consumption |  |  |
| Non-drinker | 35.7 | 32.7 |
| Light drinker | 48.2 | 50.7 |
| Heavy drinker | 16.1 | 16.6 |
| Smoking status |  |  |
| Never smoker | 49.2 | 54.3 |
| Former smoker | 25.2 | 25.1 |
| Current smoker | 25.6 | 20.6 |

Abbreviations: NHANES = National Health and Nutrition Examination Survey; NH = non-Hispanic

^1^Weighted by dust subsample survey weights to account for the complex multistage sampling design of the NHANES

^2^Weighted by interview sample survey weights to account for the complex multistage sampling design of the NHANES
